# Editorial: Global Excellence in Cardiovascular Medicine in Africa: challenges and opportunities

**DOI:** 10.3389/fcvm.2024.1479281

**Published:** 2024-10-22

**Authors:** Mahdi Garelnabi, Mpiko Ntsekhe, Anton Doubell, Masanori Aikawa

**Affiliations:** ^1^ Department of Biomedical and Nutritional Sciences, UMass Lowell Center for Population Health, University of Massachusetts Lowell, Lowell, MA, United States; ^2^ Division of Cardiology, Department of Medicine, Faculty of Health Sciences, University of Cape Town, Cape Town, South Africa; ^3^ The Division of Cardiology, Faculty of Medicine and Health Sciences, Stellenbosch University, Stellenbosch, South Africa; ^4^ Center for Interdisciplinary Cardiovascular Sciences, Division of Cardiovascular Medicine, Channing Division of Network Medicine, Brigham and Women’s Hospital and Harvard Medical School, Boston, MA, United States

**Keywords:** cardiovascular disease, Africa, cardiometabolic disorders, rheumatic heart disease (RHD), hypertension, diabetes

**Editorial on the Research Topic**
Global Excellence in Cardiovascular Medicine in Africa: challenges and opportunities

Cardiovascular medicine in Africa has seen several advances in recent years, driven by innovative approaches, international collaborations, and a growing focus on non-communicable diseases. With the rise in the utilization of the internet-based technology, remote access telemedicine platforms have been established to provide remote consultation and diagnosis, especially in rural and underserved areas in some African countries in addition mobile health applications and SMS-based interventions are being used to monitor patients’ conditions, remind them to take medications, and provide educational information about cardiovascular health (1).

Other recent advances include the increasing number of local training programs for cardiologists and other cardiovascular specialists often facilitated by the Pan African Society of Cardiology (PASCAR) and/or its affiliate national societies (2, 3). Some of these training courses are founded on partnerships with institutions in high-income countries and collaborations with international societies.

Other training opportunities have been facilitated through a growing number of successful south-south partnerships. An example is the Medtronic Foundation sponsored, PASCAR pacing training initiative which aimed to address the fact that less than a dozen African countries could offer lifesaving Pacemakers locally (4). Programs such as the Medical Education Partnership Initiative (MEPI) have been crucial in enhancing medical education and training in cardiology in many countries (5).

Conducting large-scale epidemiological studies to understand the burden and risk factors of cardiovascular disease (CVD) in African populations represent another area of recent development in the field. As can be seen from some of the published studies in this special Research Topic focusing on Africa, this area started to grow slowly, but steadily in many parts of the continent. There is certainly a great deal of improvement in data collection and health information systems to track CVD prevalence and outcomes more accurately. This data is vital for shaping public health policies and interventions. In addition, several national and regional programs were founded to focus on screening, diagnosing, and managing emerging CVD major risk factors such as hypertension, and diabetes (6) and reduce older CVD diseases such as RHD Working in collaboration with the WHO, World Heart Federation, and learning from the experience of international societies such as ESC and ACC, PASCAR and its affiliate national societies across Africa have been important in the drive to develop national, and continental policies towards the prevention and management of heart disease and its risk factors (3, 7). For example, South Africa's Strategic Plan for the Prevention and Control of Non-Communicable Diseases outlines specific targets for reducing the burden of CVD (8–10). Countries such as Nigeria and Ghana successfully implemented hypertension awareness and control programs (11, 12) that were coupled with community health workers initiatives to educate the public about cardiovascular risk factors and promote healthy lifestyles. This approach has been effective in increasing awareness and prevention efforts.

It is also important to note the enormous efforts in improving healthcare infrastructure devoted to cardiac centers of excellence in almost all African countries. Countries of noted advances include Uganda, Senegal, Cote d'Ivoire, Kenya, South Africa, Nigeria and all countries in North Africa (13, 14).

Philanthropic endeavors and other effort at investments in modern diagnostic and therapeutic equipment, including catheterization labs, echocardiography machines, and cardiac surgery facilities, are allowing a growing number of centers such as these to provide previously unavailable much needed advanced cardiac therapies and services in many African nations (15). In Egypt, Magdi Yacoub Heart Foundation transformed a public hospital in Aswan into the Aswan Heart Centre (AHC) in 2009 to serve for underserved communities in the Upper Egypt (16). The AHC has been continuously modernized to a large-scale academic medical institution that involves comprehensive clinical services (e.g., pediatric and adult cardiovascular medicine, cardiac surgery, imaging), fellowship programs, and basic science research, now serving for not only Egyptian patients but also those referred from other African nations including Mozambique, Ethiopia, Uganda, and Gambia. These enormous efforts in Africa led to overall improvement in CVD health care in the continent and decreasing the need to seek heart treatment in European countries as done in the past (14).

Despite all the above noted advances, Africa still faces several unique challenges that impact the prevention, diagnosis, and treatment of cardiovascular diseases (CVD). The continents late epidemiological transition and the slow reduction of its high burden of infectious disease, has meant that the continent now faces a double burden of both infectious diseases and non-communicable diseases (NCDs) including CVD, stretching already limited healthcare resources Increasing urbanization and lifestyle changes in many parts of Africa are leading to higher rates of hypertension, obesity, diabetes, and smoking, which are major risk factors for CVD. Persistent poverty, overcrowding and poor access to primary care has also meant that Africa also remains one of the global epicenters of Rheumatic heart disease (RHD) (17).

Unfortunately, the increased NCD and CVD burden and related risk factors has not been met by a matching rise in awareness programs, and health educational campaigns in many parts of the continent and CVD now the leading cause of morbidity and mortality in most of Africa. Additional challenges to confronting this health problem include limited adequately equipped healthcare facilities, and a grossly inadequate number of appropriately trained health care personnel for either the continent's population or burden of disease. Inadequate emergency services to handle what are often time-dependent acute cardiovascular conditions (e.g., myocardial infarction and stroke), characterize most countries across the continent including those classified as high and middle-income. Specialized training programs for Emergency Medical Technicians (EMT) to help mitigate the problem do not exist in most countries while many healthcare professionals leave Africa for better opportunities abroad, exacerbating the shortage (18). The impact of this limited access to heart health facilities and care is most evident in rural areas where disparities in health outcomes are evident (19, 20).

Finally, what cannot be overstated is wars and political disruptions in some parts of Africa which have contributed greatly to the weakening of cardiovascular medicine services undermining the public health in such areas (21). It is worth noting the ongoing war in Sudan which resulted in a complete termination of the cardiovascular medicine services in the country affecting more than 40 million Sudanese people and increasing the burden on neighboring countries who received refuges due to the ongoing war (22).

Despite these challenges, progress towards improving cardiovascular medicine in Africa remains promising and reflects a growing recognition of the importance of addressing non-communicable diseases on the continent and the political will to do so. Overcoming these challenges will require coordinated efforts from governments, healthcare providers, researchers, and the community, and continued investment in health infrastructure, training and public health initiatives to create sustainable and effective multidirectional interventions. It will also need collaborative efforts from the developed nations, and the involvement of leading heart disease professional organizations such as the American Heart Association, The American College of Cardiology, The National Lipids Association, and the European Society of Cardiology.

This Research Topic entitled, “Global Excellence in Cardiovascular Medicine in Africa” sponsored by the Frontiers in Cardiovascular Medicine highlights the advances and challenges in cardiovascular medicine across Africa, showcasing the academic excellence in the field of CVD.

Articles accepted in this collection are diverse, covering a wide range of areas of cardiovascular diseases in Africa.

RHD impacts many children across Africa. This was covered in part by an elegant review of Dr. Magdi Yacoub and his associate who reviewed the ARGI database, a hospital-based registry in a tertiary referral national center of the AHC in which all patients with the diagnosis of RHD are being included and discussed (21).

The study showed an in-depth analysis of the severity and phenotype of the disease in Egyptian patients and its progression and provided a way to compare to other regions. The ARGI database represent a good resource for other African countries. Another paper on RHD in this issue is presented behalf of the Sudanese RHD Guideline Committee. This article outlined the Sudan's rheumatic fever and RHD guidelines providing a simplified approach for countries with endemic RHD (23). In addition, the situation of anticoagulation control after mechanical heart valve replacement in low- to middle-income countries where RHD still predominates and that in developed countries substantially differs. Based on their data from 552 consecutive cases with mechanical valve replacement for RHD, Zilla et al. presented a review that outlines some best practice and guidelines for the use of mechanical valve replacement for patients with RHD discussing the international normalized ratio control in Africa and elsewhere beyond. A general commentary article by Ntsekhe and Doubell in this issue titled “*Eradicating Rheumatic Heart Disease in Africa: Have we made progress since the Drakensberg Declaration*?” summaries the state of the progress made in RHD management in Africa.

Two studies were published in this issue from Ethiopia: the first one discussing hypertension and the second presenting the incidence and predictors of recurrent acute coronary syndrome among adult patients in West Amhara. In their cross-sectional study, Gobezie et al. reported that more than half of the hypertensive patients in Ethiopia have uncontrolled BP, and diabetes mellitus. On the other hand, a study by Alamaw et al. indicated a higher incidence rate of recurrent ACS in Ethiopia, which can be explained on the light of uncontrolled major risk factors such as BP and diabetes. On the same line, Tsabedze et al. presented data for 11,860 participants from four randomized control trials on the efficacy of beta-blockers on blood pressure control and morbidity and mortality endpoints in hypertension of African ancestry. The study reported that the third-generation beta-blockers (STGBBs) can reduce the mean arterial pressure compared to other modalities, showing that these STGBBs were not associated with increased risk of stroke. Similarly reported in this issue by Musa et al. a high prevalence of hypertension among Sudanese women who had given birth to 5 fetus or more.

Other areas that were covered extensively in this issue is a study by Giliomee et al. who discussed in their review cases relevant the role of cardiovascular MRI on contextualizing tuberculous pericardial inflammation and edema as predictors of constrictive pericarditis.

The aortic diseases received extensive coverage in this issue. Meel et al. published a study involved 139 subjects with thoracic ascending aortic (TAA) aneurysms (52.5% females). The study reported an association between TAA and hypertension and HIV with considerable morbidity and mortality Indicating that a two-dimensional echocardiography and advanced strain imaging are effective methods for detecting and risk stratifying TAA aneurysms. Mvondo et al. presented an original article that discusses the pathogenesis of aortic root enlargement (ARE) in 69 patients who underwent mitral and aortic replacement. The authors reported that the association between ARE and double valve replacement (DVR) did not significantly affect the operative mortality and that ARE can be safely used whenever indications arise to reduce the occurrence of prosthesis mismatch among young patients with growth potential.

Hugo et al. in their original research article published in this issue investigated a subgroup of patients 55 years and younger of a cohort of 169 adult patients between the ages of 18 and 60, who received permanent pacemakers between 2010 and 2020 performed a retrospective audit of these young adults in South Africa. The team presented interesting data with potential utilities.

Finally, Weich et al. published an interesting original article in this special issue reporting a study that developed and tested transcatheter heart valves (THVs) that can have a reduced calcification potential. Using a juvenile sheep THV model, the authors investigated the effects of various modalities on the development of calcification and the hemodynamic function. The authors suggested that this new approach may potentially benefit younger patients.

In conclusion, this special Research Topic in Frontiers in Cardiovascular Medicine journal entitled, “Global Excellence in Cardiovascular Medicine” provides a glimpse of the challenges and opportunities in the field of cardiovascular medicine in Africa. We hope that these efforts stimulate interest in similar endeavors by the scientific community. Supporting similar work by others will greatly impact the scientific productivity by cardiovascular scientists in Africa and will eventually contribute to improving cardiovascular health care in the continent and decreasing the burden of heart diseases in Africa.
